# 2755. Activity of Aztreonam-avibactam and Carbapenem-resistant Enterobacterales (CRE) Isolates Collected in a Six-year Period (2017–2022)

**DOI:** 10.1093/ofid/ofad500.2366

**Published:** 2023-11-27

**Authors:** Mariana Castanheira, Joshua Maher, Katie Simpson, Cory Hubler, Helio S Sader

**Affiliations:** JMI Laboratories, North Liberty, Iowa; JMI Laboratories, North Liberty, Iowa; JMI Laboratories, North Liberty, Iowa; JMI Laboratories, North Liberty, Iowa; JMI Laboratories, North Liberty, Iowa

## Abstract

**Background:**

Beta-lactam/beta-lactamase inhibitor (BL/BLI) combinations and cefiderocol (FDC) have been recommended for the treatment of carbapenem-resistant Enterobacterales (CRE), but extensive comparisons of these agents are limited. We tested the activity of aztreonam-avibactam (ATM-AVI), ceftazidime-avibactam (CAZ-AVI), meropenem-vaborbactam (MEV), imipenem-relebactam (IR), all BL/BLIs, and FDC against > 500 CRE isolates collected in US hospitals during a 6-year period (2017–2022).

**Methods:**

A total of 54,576 Enterobacterales (ENT) isolates collected during 2017–2021 were susceptibility (S) tested by reference broth microdilution methods. CRE isolates displayed meropenem and/or imipenem MIC results at > 1 mg/L and were submitted to whole genome sequencing for analysis of resistance mechanisms.

**Results:**

The CRE isolates (n=511; 0.9% overall) were mainly KPC producers without metallo-beta-lactamases (n=346; 67.7%). Genes encoding MBLs and OXA-48–like enzymes alone were detected among 50 and 12 isolates, respectively. Another 12 isolates carried genes encoding NMC-A and SME. ATM-AVI inhibited 98.4% of the CRE isolates when applying ATM breakpoints for comparison. FDC, CAZ-AVI, MEV, and IR inhibited 94.7%, 89.2%, 86.7%, and 81.9% of the CRE isolates, respectively. Against class A serine-carbapenemase–producing isolates without MBLs, ATM-AVI, CAZ-AVI, FDC, and MEV demonstrated activity > 98.6%. IR inhibited 92.7% of these isolates. Except for ATM-AVI and FDC, other BL/BLI had limited activity against MBLs, ranging from 2.0% S for IR to 16% for MEV against MBL isolates. ATM-AVI inhibited 98.0% of the MBL isolates while FDC inhibited 74.0%. ATM-AVI, CAZ-AVI, IR, FDC, and MEV inhibited 94.5%, 96.7%, 90.1%, 89.0%, and 87.9% of the carbapenemase-negative isolates (n=91), respectively. Among comparators, tigecycline was the most active, inhibiting 94.7% of the CRE isolates; 84.7% of these isolates displayed a colistin intermediate MIC value of ≤ 2 mg/L.

**Conclusion:**

The new BL/BLIs and FDC displayed good activity against a large collection of CRE isolates that were mainly KPC producers from US hospitals. Against MBLs, ATM-AVI was the most active agent, followed by FDC.

**Disclosures:**

**Mariana Castanheira, PhD**, AbbVie: Grant/Research Support|Basilea: Grant/Research Support|bioMerieux: Grant/Research Support|Cipla: Grant/Research Support|CorMedix: Grant/Research Support|Entasis: Grant/Research Support|Melinta: Grant/Research Support|Paratek: Grant/Research Support|Pfizer: Grant/Research Support|Shionogi: Grant/Research Support **Joshua Maher, PhD**, AbbVie: Grant/Research Support|Affinity Biosensors: Grant/Research Support|AimMax Therapeutics, Inc: Grant/Research Support|Alterity Therapeutics: Grant/Research Support|Amicrobe, Inc: Grant/Research Support|Arietis Pharma: Grant/Research Support|Armata Pharmaceuticals, Inc: Grant/Research Support|Astrellas Pharma, Inc.: Grant/Research Support|Basilea Pharmaceutica AG: Grant/Research Support|Becton Dickinson And Company: Grant/Research Support|bioMerieux, Inc: Grant/Research Support|Boost Biomes: Grant/Research Support|Diamond V: Grant/Research Support|Fedora Pharmaceuticals, Inc: Grant/Research Support|Iterum Therapeutics plc: Grant/Research Support|Johnson & Johnson: Grant/Research Support|Kaleido Biosciences, Inc.: Grant/Research Support|Meiji Seika Pharma Co. Ltd.: Grant/Research Support|National Institutes of Health: Grant/Research Support|Pfizer Inc.: Grant/Research Support|Roche Holding AG: Grant/Research Support|Shionogi Inc.: Grant/Research Support|Summmit Therapeutics, Inc.: Grant/Research Support|Zoetis Inc: Grant/Research Support **Katie Simpson, BS**, AbbVie: Grant/Research Support **Cory Hubler, BS**, AbbVie: Grant/Research Support|Shionogi: Grant/Research Support **Helio S. Sader, MD, PhD, FIDSA**, AbbVie: Grant/Research Support|Basilea: Grant/Research Support|Cipla: Grant/Research Support|Paratek: Grant/Research Support|Pfizer: Grant/Research Support|Shionogi: Grant/Research Support

